# Synthesis and characterisation of a novel poly(2‐hydroxyethylmethacrylate)‐chitosan hydrogels loaded cerium oxide nanocomposites dressing on cutaneous wound healing on nursing care of chronic wound

**DOI:** 10.1049/nbt2.12118

**Published:** 2023-06-13

**Authors:** Jingna Luo, Weijun Liu, Qiaoling Xie, Jianshu He, Liyan Jiang

**Affiliations:** ^1^ Department of Nursing Shaanxi Provincial People's Hospital Xi'an Shaanxi China; ^2^ Department of Consumable Reagent Shaanxi Provincial People's Hospital Xi'an Shaanxi China; ^3^ Department of Nephrology The First People's Hospital of Wenling Wenling Zhejiang China; ^4^ Department of Orthopedic Surgery ChengDu Fifth People's Hospital Chengdu Sichuan China

**Keywords:** antibacterial activity, hydrogels, wounds

## Abstract

This study was designed to establish the composition of wound dressing based on poly(2‐hydroxyethylmethacrylate)‐chitosan (PHEM‐CS) hydrogels‐loaded cerium oxide nanoparticle (CeONPs) composites for cutaneous wound healing on nursing care of the chronic wound. The as‐synthesised PHEM‐CS/CeONPs hydrogels nanocomposites were characterised by using UV–visible spectroscopy, scanning electron microscopy, Fourier transform infrared spectroscopy, X‐ray diffraction, and thermo gravimetric analysis. The influence of PHEM‐CS/CeONPs hydrogels nanocomposites on the gelation time, swelling ratio, in vitro degradation, and mechanical properties was investigated. The as‐prepared PHEM‐CS/CeONPs hydrogels nanocomposites dressing shows high antimicrobial activity against *Staphylococcus aureus* and *Escherichia coli*. Similar trends were observed for the treatment of biofilms where PHEM‐CS/CeONPs hydrogels nanocomposites displayed better efficiency. Furthermore, the biological properties of PHEM‐CS/CeONPs hydrogels nanocomposites had non‐toxic in cell viability and excellent cell adhesion behaviour. After 2 weeks, the wounds treated with the PHEM‐CS/CeONPs hydrogels nanocomposite wound dressing achieved a significant closure to 98.5 ± 4.95% compared with the PHEM‐CS hydrogels with nearly 71 ± 3.55% of wound closure. Hence, this study strongly supports the possibility of using this novel PHEM‐CS/CeONPs hydrogels nanocomposites wound dressing for efficient cutaneous wound healing on chronic wound infection and nursing care.

## Graphical abstract

This study was designed to establish novel composition of wound bandages based on poly(2‐hydroxyethylmethacrylate)‐chitosan (PHEM‐CS) hydrogels‐loaded cerium oxide nanoparticle (CeONPs) composites. This novel PHEM‐CS/CeONPs hydrogels nanocomposites dressing strongly supports for efficient cutaneous wound healing and nursing care on chronic wound infection.

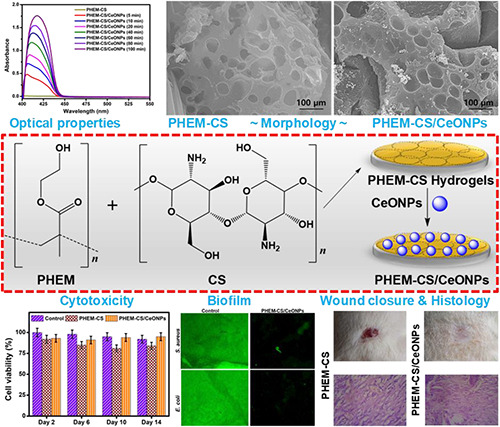

## 1 INTRODUCTION

The major difficulty in the management of wound care is its infection. When the cell is damaged by physiochemical forces, such as cuts, scalds, or burns, injuries can be restored to the stability of the skin tissue structure [1]. Human skin can regenerate itself, but because of its long‐term repair strategy and the possibility of consequences like swelling and subsequent injuries, especially for large full‐thickness wounds, it has limited availability in the creation and advancement of wound dressings [2]. Insufficient care and repair of the wound may lead to infection, other pathological reactions, and even death [3]. Furthermore, provides a high level of moisture in the wound area, which speeds up regeneration and tissue formation. Since their flexibility, comparable to the original extracellular matrix, controllable physical and chemical properties, and ease of covering unusual skin infections, hydrogel‐based dressings with injectable qualities have become potential possibilities for wound dressings [4]. Therefore, the creation of injectable polymer nanocomposite treatments with several functions holds promise for the treatment of wounds [5]. In both clinical and experimental settings, chronic healing has been studied to facilitate the healing process and to inhibit the factors preventing the occurrence of the phenomenon [6]. It is essential that a patient with a wound infection, whether cast or in traction, be monitored carefully to prevent complications as the wound heals. In assessing and caring for patients with a chronic wounds, the nursing assessment should take a holistic approach, since wound infections are not always the most serious injury they may have sustained. In modern medical practice, external dressings are often used to guard against the entry of bacteria into the wound and provide a temporary solution for damaged barriers [7, 8]. For this reason, the design and development of polymer‐based nanocomposite dressings with therapeutic agents incorporated within them can play a vital role in nursing care for healing skin wounds [4, 9, 10].

Metal nanoparticles have been widely employed in the biomedical and pharmaceutical industries [11]. Metal oxides, such as cerium oxide (CeO_2_), are thought to be efficient antioxidants that can speed up local healing [12]. Additionally, CeO_2_ has shown some other prospective medicinal uses, such as neuroinflammation, nuclear safety, and defense versus apoptosis brought on by hydrogen peroxide [13]. CeO_2_ nanoparticles (CeONPs) have recently been discovered to act as radical scavengers, removing excess reactive oxygen and nitrogen species such as superoxide anion, hydroxyl radicals, hydrogen peroxides, and nitric oxide radicals, piqued interest in their potential biomedical fields [14]. CeONPs, in notably, have been shown to speed up the healing of skin wounds by increasing the proliferation and metastasis of key epidermis cells [12, 15]. Meanwhile, polymer hydrogels are ideal for nanoparticle incorporation due to their possibility to control Cerium release and other excellent properties.

Hydrogel wound dressings have good tissue adhesion, stretchability, and self‐healing properties among the wound dressings currently studied. The majority of hydrogel wound dressing studies focus on their multifunctionality while neglecting the challenge of wound movement [16]. Poly(hydroxyethylmethacrylate) (PHEM), a chemical hydrogel, has been widely explored in the past decades on its chemical–physical stability and mechanical characteristics, among a wide spectrum of innovative polymer composites [17]. This biodegradable gel has been employed in a range of tissue engineering, including contact and intraocular lenses, vascular prosthesis, drug delivery systems, and soft‐tissue regeneration. When PHEM polymerises in water, it produces a specific morphology of hydrogels largely dependent on its molecular weight and extent of cross‐linking [18]. Water‐insoluble PHEM chains with molecular weights >2800 Da (as in this study) dissociate from water to create a 3D network of polymeric particles with interconnecting holes. Chitosan (CS), a natural amino homogenous linear polysaccharide generated from deacetylated chitin, is made up of glucosamine and N‐acetylglucosamine units joined by the β‐(1–4) glycosidic linkage [19]. Because of its higher reactivity and solubility than chitin, this substance with free amino groups does not pollute the environment. Hydrogels can be made simply from chitin and CS. Healing, antibacterial, antifungal, and chelating capabilities would have been included in addition to those described. CS is also bioadhesive, which promotes retention at the insertion point due to its positive charges at physiological pH [20]. The fact that CS is abundant in nature, produced cheaply, and has ecological significance is crucial. As a result, CS was used in the healthcare and biopharmaceutical industries, as well as in the hydrogel matrix. Following this lead, CeONPs were first integrated into poly(2‐hydroxyethylmethacrylate)‐chitosan (PHEM‐CS) hydrogels for wound healing via a simple evaporation‐induced self‐assembly approach.

Herein, we report the synthesis and characterisation of a novel PHEM‐CS hydrogels‐loaded CeONPs composites for cutaneous wound healing in nursing care of the chronic wound. The optical properties, morphology, surface analysis, gelation mechanism, swelling ratio, degradation behaviour, and mechanical properties of the PHEM‐CS/CeONPs hydrogels nanocomposites were systematically studied. The antibacterial and antibiofilm activities of the resulting PHEM‐CS/CeONPs hydrogels nanocomposites against Gram‐positive and Gram‐negative bacteria were evaluated. Cell viability experiments were conducted to verify the PHEM‐CS/CeONPs hydrogels nanocomposites with good biocompatibility. Finally, hydrogel nanocomposites were investigated in a mouse model of full‐thickness skin defect infected by the PHEM‐CS/CeONPs to encourage the development of blood vessels and epithelial tissue and to further increase wound healing.

## 2 MATERIALS AND METHODS

### 2.1 Materials

2‐hydroxyethyl methacrylate, ethylene glycol dimethacrylate (EGDMA) Azobis isobutyronitrile (AIBN), and Chitosan (average viscosity 5,270,000 Da and DD 86%) were obtained from Sigma‐Aldrich. Ce(NO_3_)_3_·6H_2_O (99%) was purchased from Sinopharm Chemical Reagent Co., Ltd, Beijing, China. All other reagents used in the experiment were of analytical grade.

### 2.2 Fabrication of PHEM‐CS/CeONPs

CeONPs were made by hydrothermal technique from Ce(NO_3_)_3_ hydrolysis in an acetate–acetic acid buffer solution. In a nutshell, 10 g sodium acetate, 10 mL acetic acid, and 2.17 g cerium nitrate were dissolved in water and diluted to 85 mL. A 100 mL Teflon‐lined stainless‐steel autoclave was then used to heat the solution to 200°C for 24 h. The materials were collected, five times rinsed with distilled water, and then discarded in water for use after cooling. The hydrogels were created by polymerising the HEMA monomer. In a nutshell, EGDMA was combined with the HEMA monomer at a ratio of 3% w/w and 1% w/w, respectively, to act as a crosslinker and an AIBN initiator. The copolymer reaction was carried out for 8 h at 70°C, proceeded by ethanol cleaning to eliminate any residual molecules. PHEM (8% w/v) and CS (1% w/v) aqueous solutions were made by dissolving in deionised water and 3% v/v glacial acetic acid, respectively. For the preparation of the PHEM‐CS matrix containing a precise amount of CeONPs was mixed thoroughly. For three continuous cycles, the correct amount of this mixture was put into Petri dishes, then frozen at 20°C for 18 h, and then cooled at room temperature (30 min).

### 2.3 Gelation time, swelling ratio, in vitro degradation

The test‐tube inverted method was used to measure the gelation time. After being transferred to a glass vial, the freshly made PHEM‐CS hydrogels and PHEM‐CS/CeONPs hydrogel nanocomposites solution (3 mL) was heated in a water bath at 37°C with gentle shaking to facilitate gelation. The vials were then put in a bath at a regulated temperature after that. By horizontally inverting the vials every minute, the sol‐gel transition time was calculated. The period during which the gel did not flow was referred to as the gelation time.

All of the dry scaffolds were measured and labelled with the number *W*_0_. The scaffolds were then placed for 36 h in phosphate buffer saline (PBS) at pH 7.4 at 37°C. The scaffolds were taken down after 36 h, and any water that had been adsorbed was cleaned off with filter paper. The scaffolds' wet weights were measured and recorded as *W*_*w*_. The following formula was used to calculate the swelling ratio: swelling ratio = *W*_*w*_ − W_0_/W_0_.

Equally weighed scaffolds were placed in media containing lysozyme at a concentration comparable to the blood circulation levels (10 mg/L), and the mixture was incubated for 3 days at 37°C. After 24, 48, and 72 h of incubation, the scaffolds were rinsed in water to remove ions from the surface and dried. The initial weight of the scaffolds was noted as *W*_0_. *W*_*t*_ was used to denote the dry weight. The following formula was used to determine the degradation of the scaffold:

$$Degradation\;ratio\;\left(\%\right)=W_{0}\;–\;W_{t}/W_{0}$$

### 2.4 Mechanical properties

The tensile strength and degree of elongation break of PHEM‐CS hydrogels and PHEM‐CS/CeONPs hydrogels nanocomposites blend has been measured using a tensile test machine (EZ‐LX, Shimadzu). PHEM‐CS hydrogels and PHEM‐CS/CeONPs hydrogels nanocomposites were cut into 10 × 0.5 cm. The clamping distance was adjusted to 50 mm, with a 5 mm/min stretching speed. Before the investigation, the membrane thickness was measured using a digital Vernier calliper.

### 2.5 Characterisations

A Perkin Elmer Lambda 35 UV‐Vis spectrophotometer was used to measure the production of CeONPs in the colloidal solution and the presence of PHEM‐CS hydrogels. Scanning electron microscopy (SEM) was used to examine the surface morphology of PHEM‐CS hydrogels and PHEM‐CS/CeONPs hydrogels nanocomposites using a LEO SUPRA 55 (Carl Zeiss AG) with 10 kV voltages. Snap freezing was used to create the PHEM‐CS hydrogels and PHEM‐CS/CeONPs hydrogel nanocomposites, which were then coated in gold using an ion sputter coater (Hitachi S3400N) before being lyophilised. The Fourier transform infrared spectroscopy (FTIR) bands were obtained by capturing bands with a resolution of 4 cm^−1^ between 4000 and 400 cm^−1^ (Nicolet‐6700, Thermo Electron). The samples were dried for 48 h in a dry steriliser oven to eliminate the absorbed water from the PHEM‐CS hydrogels and PHEM‐CS/CeONPs hydrogel nanocomposites before they were ready for analysis. The X‐ray diffraction (XRD) patterns of PHEM‐CS hydrogels and PHEM‐CS/CeONPs hydrogels nanocomposites were examined using a (Panalytical's X'Pert Pro) with cu K radiation (*λ* = 1.54) operated at 45 kV voltage and 40 mA in the scan range of 10–80° (2θ) at ambient temperature. The thermal stability of PHEM‐CS hydrogels and PHEM‐CS/CeONPs hydrogel nanocomposites were evaluated using a TA 5000/SDT 2960 thermal analyser (Zurich) at a heating rate of 10°C/min under N_2_.

### 2.6 Antibacterial and biofilm activity

Using *Staphylococcus aureus* ATCC 25923 and *Escherichia coli* ATCC 25922, the antibacterial activity of PHEM‐CS hydrogels and PHEM‐CS/CeONPs hydrogel nanocomposites was assessed. Each group bacterial suspension was divided into 100 μL portions and spread out on a solid agar medium. Gentamicin disc (10 μg) was used as control. To obtain a bacterial culture with a particular concentration, both bacteria were grown in nutrient broth at 37°C for 24 h. To make the bacterial culture preparations, they were diluted with sterile PBS to a concentration of 6 × 10^8^ CFU/mL. 1 mL test bacterial suspension was homogeneously injected into 150 μL sterile agar medium. In the end, the prepared solid agar culture dishes were covered with a sealing membrane and kept at 37°C for 12 h of culture. To assess the antimicrobial property of the hydrogel dressings, the diameter of the inhibition zone was assessed. Then, according to their growth rates, the diluted cell solution (100 μL) was inoculated into each well of a 96‐well plate. *Staphylococcus aureus* was kept shaking at 100 rpm, 37°C, and 50 rpm, 25°C, respectively, due to differences in the rate of biofilm development, whereas *E. coli* were incubated at 37°C without shaking. Before treating the biofilm, the used medium was removed, and the adherent cells were removed by gently washing the biofilm with PBS. The biofilm was then shielded for 24 h with 50 μL of PHEM‐CS hydrogels and PHEM‐CS/CeONPs hydrogel nanocomposites.

### 2.7 Cell viability assay

To test the in vitro cytotoxicity of PHEM‐CS hydrogels and PHEM‐CS/CeONPs hydrogel nanocomposites, murine NIH‐3T3 fibroblast cells were used. The PHEM‐CS hydrogels and PHEM‐CS/CeONPs hydrogels nanocomposites samples were sterilised at 60°C for 24 h. And then dissolved in 2 mL DMEM media to obtain a 50 mg/mL precursor solution. After the cells had adhered to the wall, the sample washing solution was used to replenish the 100 μL of culture media. The plate was incubated at 37°C for 2 h in an incubator with 5% CO_2_. Finally, a microplate reader was used to test the absorbance at 450 nm on the 96‐well plate. The viability of the cells was assessed at different intervals on days 2, 6, 10, and 14.

### 2.8 In vivo wound healing studies

All animal protocols in this study were approved by the Animal Care and Use Committee on the Ethics of Animal Experiments at the First People's Hospital of Wenling (approval number: FHW201406). In vivo wound healing investigations were performed on Sprague‐Dawley female mice weighing 25 g. Mice were housed in a ventilated and clean animal house with a temperature of 25°C, relative humidity of 50%, and a 12‐h light/dark cycle. All mice were randomly divided into two groups: the PHEM‐CS hydrogels group and PHEM‐CS/CeONPs hydrogels nanocomposites group. The 8 mice were randomly divided into the following sampling points: 2, 6, 10, and 14 days and each sampling point had four mice (*n* = 4). After grouping, all mice underwent the following operations under aseptic conditions: (i) all mice were anaesthetised by intraperitoneal injection of 10% chloral hydrate solution with 3.33 mL/kg. (ii) The hair on the back of all mice was removed to facilitate the following operations. (iii) Two round shapes of the full‐thickness wound (1 × 1 cm^2^ diameter) were created in the middle of the back of each mouse. Rats were housed in cages and given equal amounts of food and water at a steady temperature. On days 2, 6, 10, and 14, after surgery, optical pictures of the wounds were taken. The samples were taken for optical imaging, and ImageJ was used to calculate the wound area quantitative statistics.

### 2.9 Histological studies

To adhere the PHEM‐CS hydrogels and PHEM‐CS/CeONPs hydrogel nanocomposites to the rat wound sites, they were cut into circles. Rats were put to death for histological examination after 2, 6, 10, and 14 days of recovery respectively. The skin wound tissue was removed, then sliced into small sections (4 μm) and preserved in a PBS solution containing 4% formaldehyde solution at room temperature for at least 24 h. Finally, the sections were stained with haematoxylin and eosin (H&E) using a microscope.

### 2.10 Statistical analysis

The results were expressed by mean ± standard deviation (SD). For biological studies, at least three replicates were involved and final results expressed as the mean ± SD. Unpaired Student's *t*‐test was used to determine *p* value. The significance criterion for calculating the statistical differences between samples was set at *p* < 0.05.

## 3 RESULTS AND DISCUSSION

### 3.1 Gelation time, swelling ratio, and in vitro degradation

**FIGURE 1:**
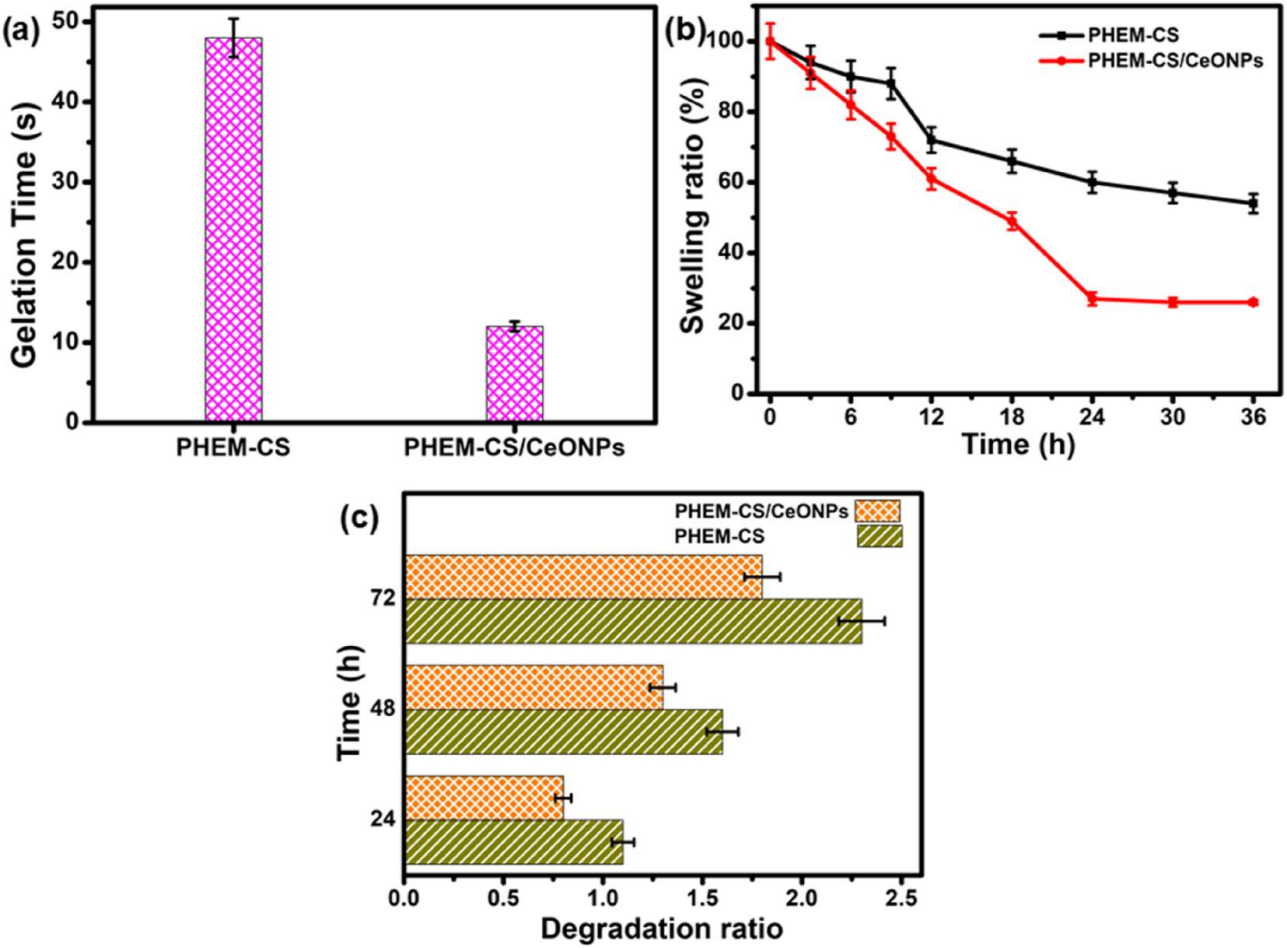
(a) Gelation time, (b) swelling ratio, and (c) In vitro degradation studies of PHEM‐CS hydrogels and PHEM‐CS/CeONPs hydrogels nanocomposites. Results are expressed as mean ± SD (*n* = 3), *p* < 0.05. CeONPs, cerium oxide nanoparticles; PHEM‐CS, poly(2‐hydroxyethylmethacrylate)‐chitosan.

Figure 1a illustrates the gelation time of PHEM‐CS hydrogels and PHEM‐CS/CeONPs hydrogels nanocomposites. With the addition of CeONPs to the PHEM‐CS hydrogels, the required time for gelation decreases [21]. Gelation time for PHEM‐CS hydrogels and PHEM‐CS/CeONPs hydrogels nanocomposites was 48 ± 2.4 and 12 ± 0.6 min respectively. The PHEM‐CS hydrogels solution with CeONPs took <12 min to become a gel whereas with PHEM‐CS hydrogels the gelation time was more than 48 min. CeONPs‐contained PHEM‐CS hydrogel solutions were prepared in situ by the reduction of cerium ion (Ce^3+^) to form CeONPs via a reducing reaction mainly with the hydroxyl and amine groups on PHEM‐CS hydrogels [22].

The swelling ratio was used to assess the ability of nanomaterials to absorb and retain water, as well as their stability [23]. A good swelling hydrogel can efficiently offer adequate fluid absorption and a microenvironment at the wound site, both of which are required for effective wound treatments. The swelling ratios of PHEM‐CS hydrogels and PHEM‐CS/CeONPs hydrogels nanocomposites were plotted as a function of time in Figure 1b. Within 24 h, all samples reached equilibrium swelling, and the swelling ratio increased when CeONPs were added to the PHEM‐CS hydrogels [24]. Figure 1c showed the in vitro degradation of PHEM‐CS hydrogels and PHEM‐CS/CeONPs hydrogels nanocomposites were carried out by incubating in PBS 7.4. The PHEM‐CS/CeONPs hydrogels nanocomposites at 24, 48, and 72 h showed less degradation by the enzyme when compared to PHEM‐CS hydrogels. The incorporation of CeONPs in the PHEM‐CS hydrogels decreased its biodegradation suggesting the availability of PHEM‐CS/CeONPs hydrogels nanocomposites for a longer period till the new bone tissue in growth proceeds [25].

### 3.2 Mechanical properties of PHEM‐CS/CeONPs hydrogels nanocomposites

**FIGURE 2:**
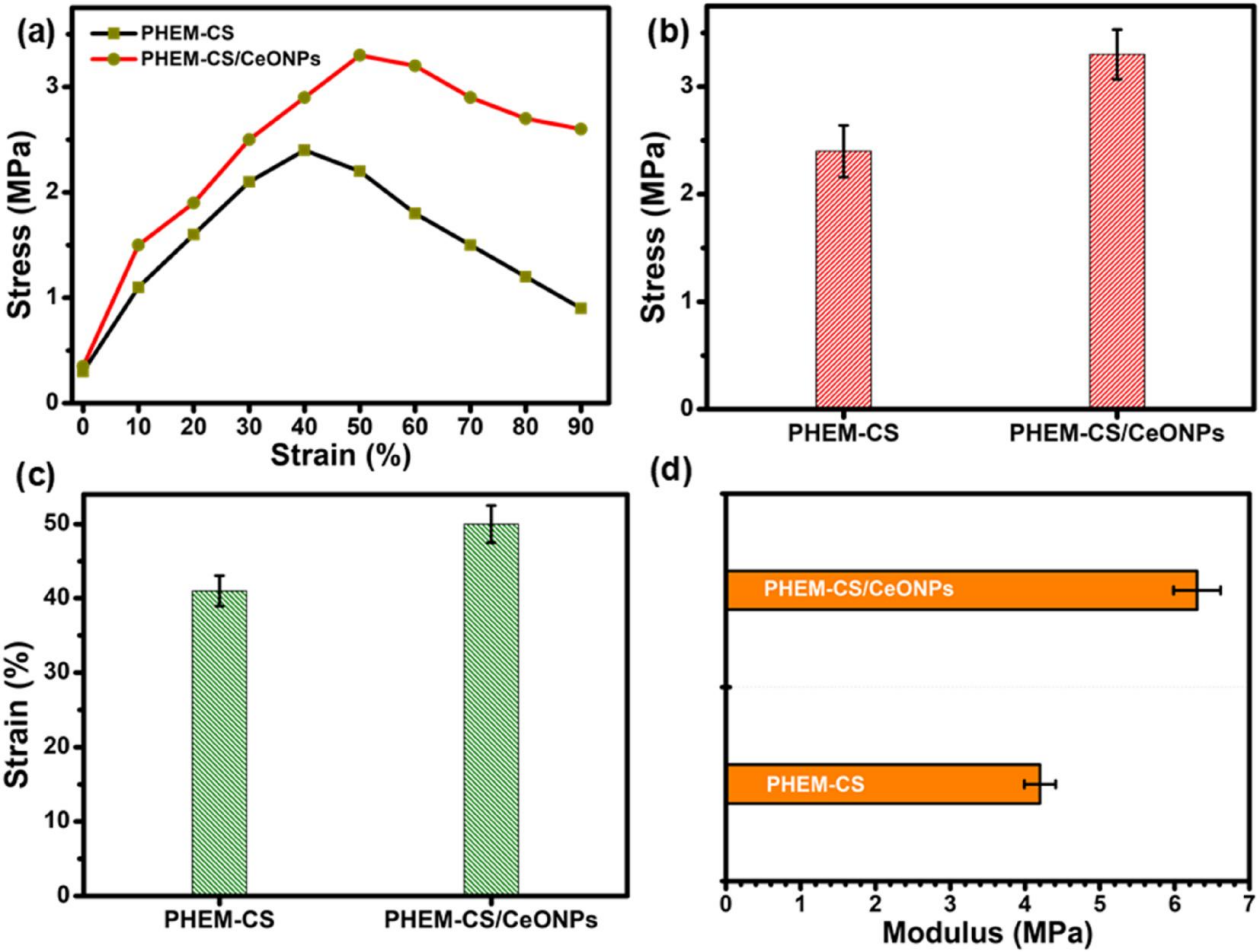
Mechanical properties comparison of (a) stress‐strain curve, (b) tensile strength, (c) elongation, and (d) tensile modulus of PHEM‐CS hydrogels and PHEM‐CS/CeONPs hydrogels nanocomposites. Standard stress‐strain curves (*n* = 0.1). Results are expressed as mean ± SD (*n* = 3), *p* < 0.05. CeONPs, cerium oxide nanoparticles; PHEM‐CS, poly(2‐hydroxyethylmethacrylate)‐chitosan.

The mechanical properties of the hydrogel nanocomposite are important for effective dressing applications. To achieve the desired wound healing, wound dressings must be robust and flexible [26]. The mechanical properties (tensile strength, maximum elongation, and tensile modulus) of PHEM‐CS hydrogels and PHEM‐CS/CeONPs hydrogels nanocomposites are illustrated in Figure 2. The PHEM‐CS/CeONPs hydrogels nanocomposites exhibited much higher ductility and the positive effect of CeONPs on the tensile properties of the PHEM‐CS/CeONPs hydrogels nanocomposites was well reflected in the tensilestress–strain curves as shown in Figure 2. The addition of CeONPs to the PHEM‐CS hydrogels had a varied influence on the tensile strength and total elongation, it was discovered [27]. The tensile strength of the PHEM‐CS hydrogels and PHEM‐CS/CeONPs hydrogels nanocomposites was 2.4 ± 0.24 and 3.3 ± 0.23 MPa, respectively (Figure 2a,b), which was suitable for tissue covering the wound. PHEM‐CS/CeONPs hydrogels nanocomposites were stretchable in the presence of CeONPs used, with a higher elongation at break and tensile strength at fracture than PHEM‐CS hydrogels. Because of their crosslinking capabilities, CeONPs in PHEM‐CS hydrogels significantly increased the tensile strength of nanocomposite films [28].

The as‐prepared PHEM‐CS hydrogels and PHEM‐CS/CeONPs hydrogels nanocomposites exhibited an elongation in the range from 40% to 50% at the wound points. With the addition of CeONPs, the tensile strength improved. The tensile modulus value matched the stress test perfectly. To minimise breaking due to exudate absorption and the patient's motions, hydrogel dressings must be able to tolerate the applied force on the wound site and have a greater threshold for swelling or motion‐induced distortion [30]. As a result, the PHEM‐CS/CeONPs hydrogel nanocomposites had a lower strength property but a greater tensile modulus and elongation than the PHEM‐CS hydrogels. To apply PHEM‐CS/CeONPs hydrogel nanocomposites to diverse types of wound surfaces, flexibility would be a key factor.

### 3.3 Optical properties of PHEM‐CS/CeONPs hydrogels nanocomposites

**FIGURE 3:**
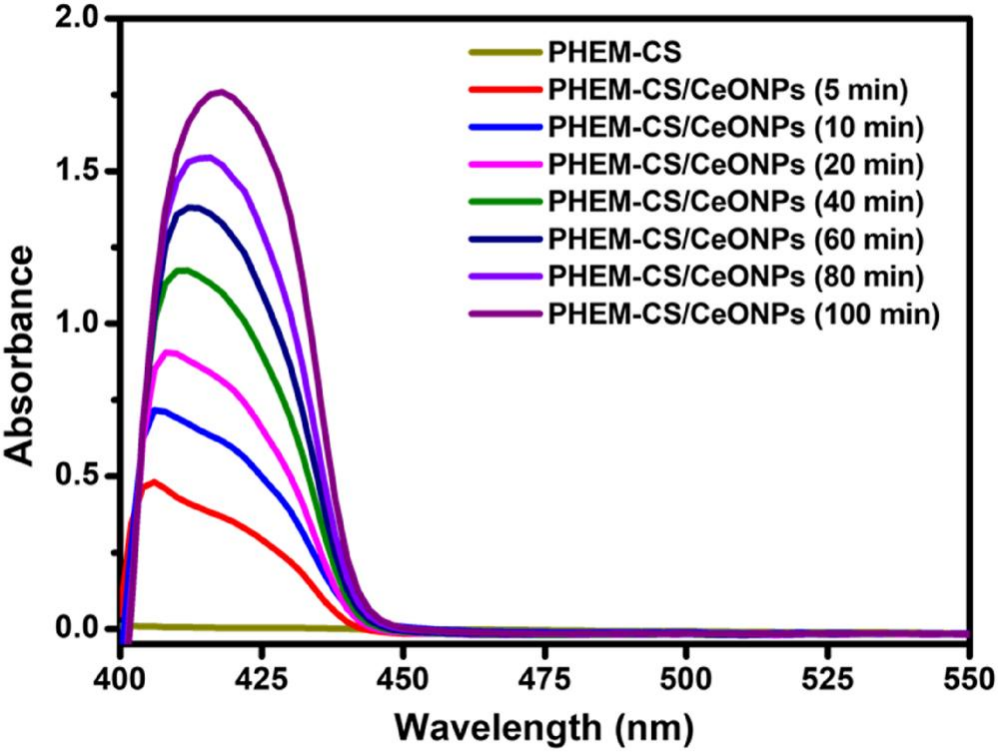
UV‐visible studies of PHEM‐CS hydrogels and in the presence of CeONPs with increasing reaction time at 5, 10, 20, 40, 60, 80, and 100 min. CeONPs, cerium oxide nanoparticles; PHEM‐CS, poly(2‐hydroxyethylmethacrylate)‐chitosan.

The existence of CeONPs in the PHEM‐CS hydrogels networks was tested by using UV‐visible spectral analysis (Figure 3). There is no peak between 400 and 450 nm was observed in PHEM‐CS hydrogels. Generally, CeONPs exhibit versatile and changeable optical property that depends on their size and shape [30]. At 5 min of reduction time, the resonance band was found between 400 and 450 nm, indicating the synthesis of CeONPs. A significant improvement in the absorption peak (*λ*_max_ = 408–425 nm) was observed for PHEM‐CS/CeONPs hydrogels nanocomposites due to the resonance effect [31]. Because of differences in nanoparticle size, the intensity of the peak increased from 10 to 100 min with considerable redshift shifts in the maximum absorption. The CeONPs plasma intensity band at 424 nm showed no discernible change after 100 min, indicating that the reduction reaction was complete at that time. The development of CeONPs in the PHEM‐CS hydrogels was confirmed by this spectrum investigation.

### 3.4 Morphology of PHEM‐CS/CeONPs hydrogels nanocomposites

**FIGURE 4:**
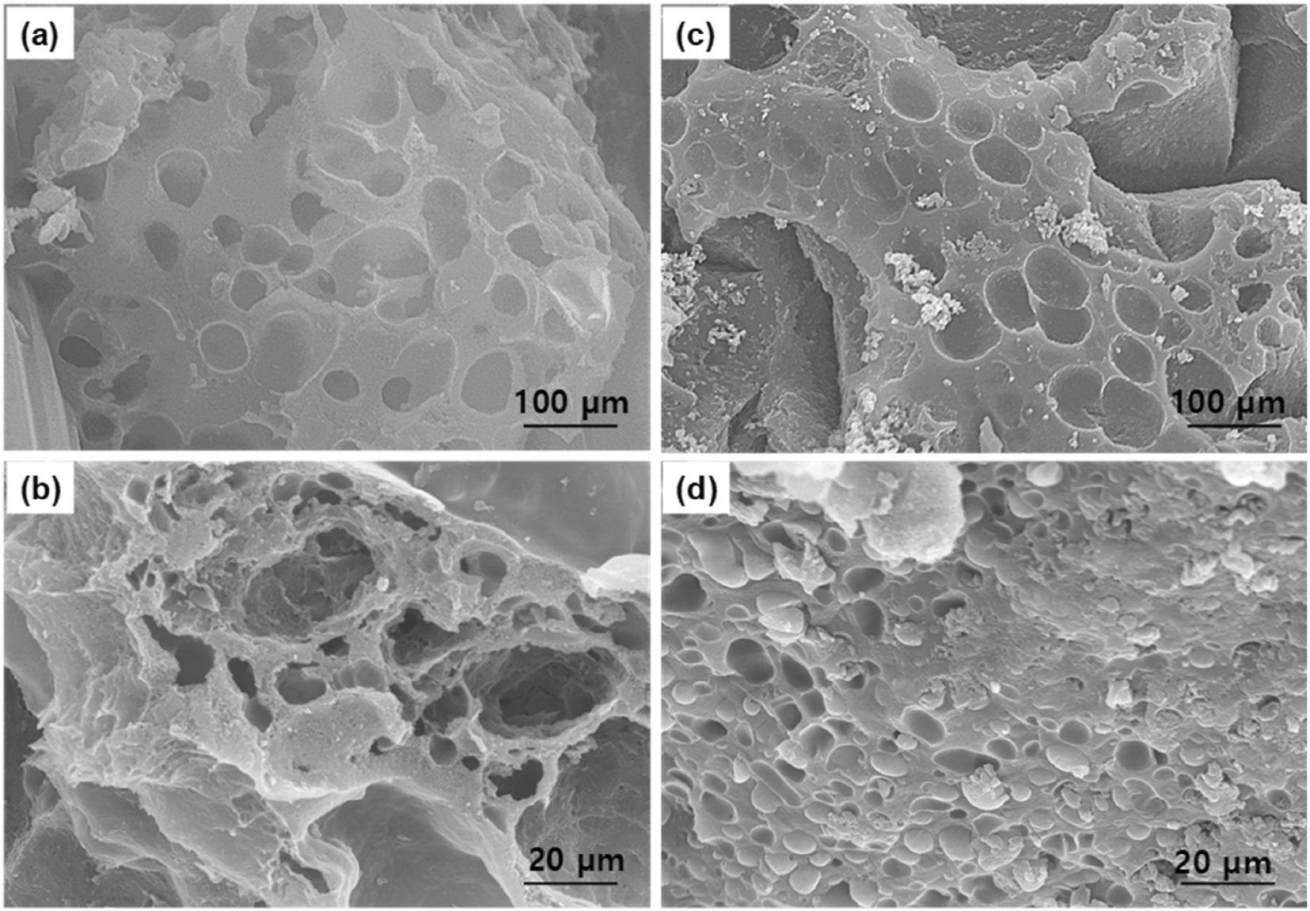
SEM porous morphology with different magnifications of (a, b) PHEM‐CS hydrogels and (c, d) PHEM‐CS/CeONPs hydrogels nanocomposites. CeONPs, cerium oxide nanoparticles; PHEM‐CS, poly(2‐hydroxyethylmethacrylate)‐chitosan; SEM, scanning electron microscopy.

SEM analysis proves the morphological differences in both stages of the hydrogel. The two types of PHEM‐CS hydrogels without CeONPs and PHEM‐CS hydrogels with CeONPs are illustrated in Figure 4. The PHEM‐CS hydrogels and PHEM‐CS/CeONPs hydrogels nanocomposites show a three‐dimensional network with well‐defined pores with a diameter ranging between 20 and 80 μm [32]. Micropores and crosslinked networks were consistently distributed on the surface of the PHEM‐CS hydrogels. It was well known that interconnected pore structures may speed up water transport through the PHEM‐CS hydrogels matrix and play a significant role in the rapid swelling of hydrogels (Figure 4a,b) [33]. SEM images of PHEM‐CS/CeONPs hydrogels nanocomposites show morphology‐like particles; the CeONPs within the PHEM‐CS hydrogel network caused an increased porous structure in the hydrogels nanocomposites (Figure 4c,d) [34]. When the Ce^3+^ ions were absorbed, they were able to diffuse through the pores thanks in large part to the PHEM‐CS/CeONPs hydrogel nanocomposites with the appropriate permeability. It can be safely assumed that the addition of CeONPs into the PHEM‐CS hydrogel tends to affect the properties of the gel significantly.

### 3.5 Surface analysis and thermal stability of PHEM‐CS/CeONPs

**FIGURE 5:**
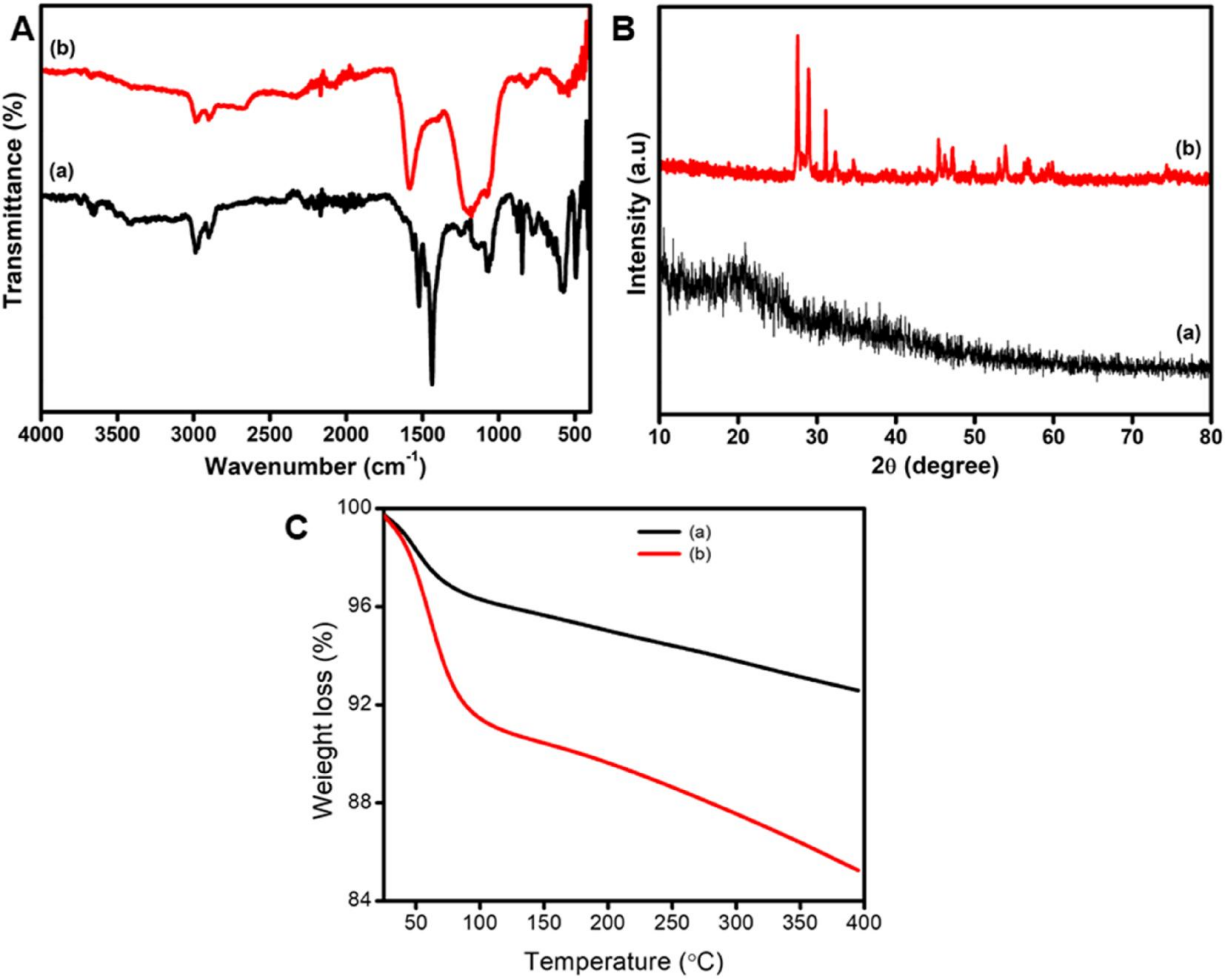
(a) FTIR spectra, (b) XRD pattern, and (c) TGA analysis of PHEM‐CS hydrogels and PHEM‐CS/CeONPs hydrogels nanocomposites. CeONPs, cerium oxide nanoparticles; FTIR, fourier transform infrared spectroscopy; PHEM‐CS, poly(2‐hydroxyethylmethacrylate)‐chitosan; TGA, thermo gravimetric analysis; XRD, X‐ray diffraction.

The main characteristic FTIR absorption bands of cross‐linked PHEM‐CS hydrogels and PHEM‐CS/CeONPs hydrogels nanocomposites are presented in Figure 5a. The broad strong peak between 3000 and 3600 cm^−1^ in the FTIR spectra of CS is ascribed to overlapping O–H and N–H stretching vibrations. The C–H of the methyl (–CH_3_) groups and alkyl (–CH_2_–) chains are responsible for the peak at 2875 cm^−1^ [35]. The cross‐linked PHEM‐CS hydrogels FTIR spectra show a peak shift from 3000 to 3700 cm^−1^. This shift is assumed to be produced by the use of acetic acid as a solvent, which protonates the amine group [36]. The PHEM‐CS hydrogels product has a CS backbone with ‐COO functional groups on the side chains, as demonstrated by novel peaks at 1576 and 2246 cm^−1^ respectively. The highly intense characteristic band at 1576 cm^−1^ is attributable to carboxylate anion C=O asymmetric stretching, which is corroborated by another sharp peak at 1448 cm^−1^, which is connected to the ‐COO symmetric stretching mode [37]. The absorption peaks of the cross‐linked PHEM‐CS hydrogels with CeONPs were 3418–3395 cm^−1^ (wide peaks owing to H‐bond formation) for the –OH hydroxyl group, 2909–2881 cm^−1^ for the alkyl group, and 1099 cm^−1^ for the C–O stretching of the ether group. Figure 5b shows the XRD patterns of PHEM‐CS hydrogels and PHEM‐CS/CeONPs hydrogels nanocomposites. The diffraction peaks of the PHEM‐CS hydrogels include a large diffraction peak 2θ that appears at 23.2° and 31.5° [38]. The diffraction peaks associated with PHEM‐CS hydrogels are very weak indicating their low amorphous nature. The incorporated CeONPs remain intact in a polymeric network of PHEM‐CS/CeONPs hydrogels nanocomposites, which is indicated by the presence of characteristic peaks of CeONPs in the diffraction pattern of CeONPs loaded PHEM‐CS hydrogels.

The thermo gravimetric analysis (TGA) curves of PHEM‐CS hydrogels and PHEM‐CS/CeONPs hydrogels nanocomposites are presented in Figure 5c. The TGA spectra of PHEM‐CS hydrogels indicated that the initial weight loss is due to a loss in sample moisture content and is seen in the temperature range of 75–120°C. The decomposition of the PHEM‐CS hydrogels is responsible for the highest weight loss (6.28%), which is noted in the temperature range of 390°C. Similar trends of weight loss in two stages are shown on the thermogram of PHEM‐CS/CeONPs hydrogel nanocomposites: In the temperature range of 90–120°C, an initial decomposition of around 8.95% weight loss is seen, which may be caused by the removal of water from PHEM‐CS/CeONPs hydrogel nanocomposites. Due to the disruption of PHEM‐CS/CeONPs cross‐linked polymeric structure, the primary disintegration of these hydrogel nanocomposites occurs at a temperature of 390°C and 15.2% weight loss. The PHEM‐CS hydrogel might be effectively useful for cerium‐releasing wound dressing applications.

### 3.6 Antibacterial and biofilm

The antimicrobial properties of CS make it widely used as an antimicrobial agent either alone or combined with other natural polymers [39]. We examined the antibacterial activity of PHEM‐CS hydrogels and PHEM‐CS/CeONPs hydrogels nanocomposites for the zone of inhibition against *E. coli* and *S. aureus* bacterial species since the objective of this research was to produce scaffolds that impart antimicrobial properties through CeONPs incorporation (Table 1). In actuality, the scaffold pores or porous structure is more likely to be an ideal setting for bacterial activities [40]. The zone inhibition of the PHEM‐CS/CeONPs hydrogels nanocomposites against *E. coli* and *S. aureus* was determined to be 19.5 ± 2.39 and 14.8 ± 1.14 mm respectively. The PHEM‐CS hydrogels (without CeONPs) had a reduced zone of inhibition against *E. coli* and *S. aureus*, measuring 11.6 ± 1.28 and 8.3 ± 0.63 mm, respectively, which could be owing to the presence of CS, which has natural antibacterial action. Whereas, all prepared hydrogels nanocomposites have acceptable antibacterial effects on both *E. coli* and *S. aureus* (ranging 8–19.5 mm) with lower than Gentamicin as a control (21 ± 1 and 22 ± 1 mm respectively). The increased zone of inhibition for the PHEM‐CS hydrogels with CeONPs has attributed to cerium antibacterial activity, according to the findings [41]. Quantitative results also demonstrated that the PHEM‐CS/CeONPs hydrogels nanocomposites group had the highest bacterial killing activity of both types of bacteria. CeONPs against *E. coli* demonstrated that the bactericidal activity of nanoparticles increases with a decrease in particle size. In particular, CeONPs have been reported to exhibit antibacterial activity through several mechanisms [42, 43, 44]. Accordingly, the antibacterial efficacy of PHEM‐CS hydrogels and PHEM‐CS/CeONPs hydrogels nanocomposites against the tested pathogens was considerable and statistically significant. As a result, the porous structure, pore diameters, wettability, and non‐toxicity of the PHEM‐CS/CeONPs hydrogels nanocomposites created using this technique could be used effectively in biomedical applications.

**TABLE 1:** Antibacterial activity of PHEM‐CS hydrogels and PHEM‐CS/CeONPs hydrogels nanocomposites towards *Staphylococcus aureus* and *Escherichia coli*.

Samples	Diameter zone of inhibition (mm)	
	*S. aureus*	*E. coli*
Control	21.0 ± 1.0	22.0 ± 1.0
PHEM‐CS	8.3 ± 0.63	11.6 ± 1.28
PHEM‐CS/CeONPs	14.8 ± 1.14	19.5 ± 2.39

Abbreviations: CeONPs, cerium oxide nanoparticles; PHEM‐CS, poly(2‐hydroxyethylmethacrylate)‐chitosan.

**FIGURE 6:**
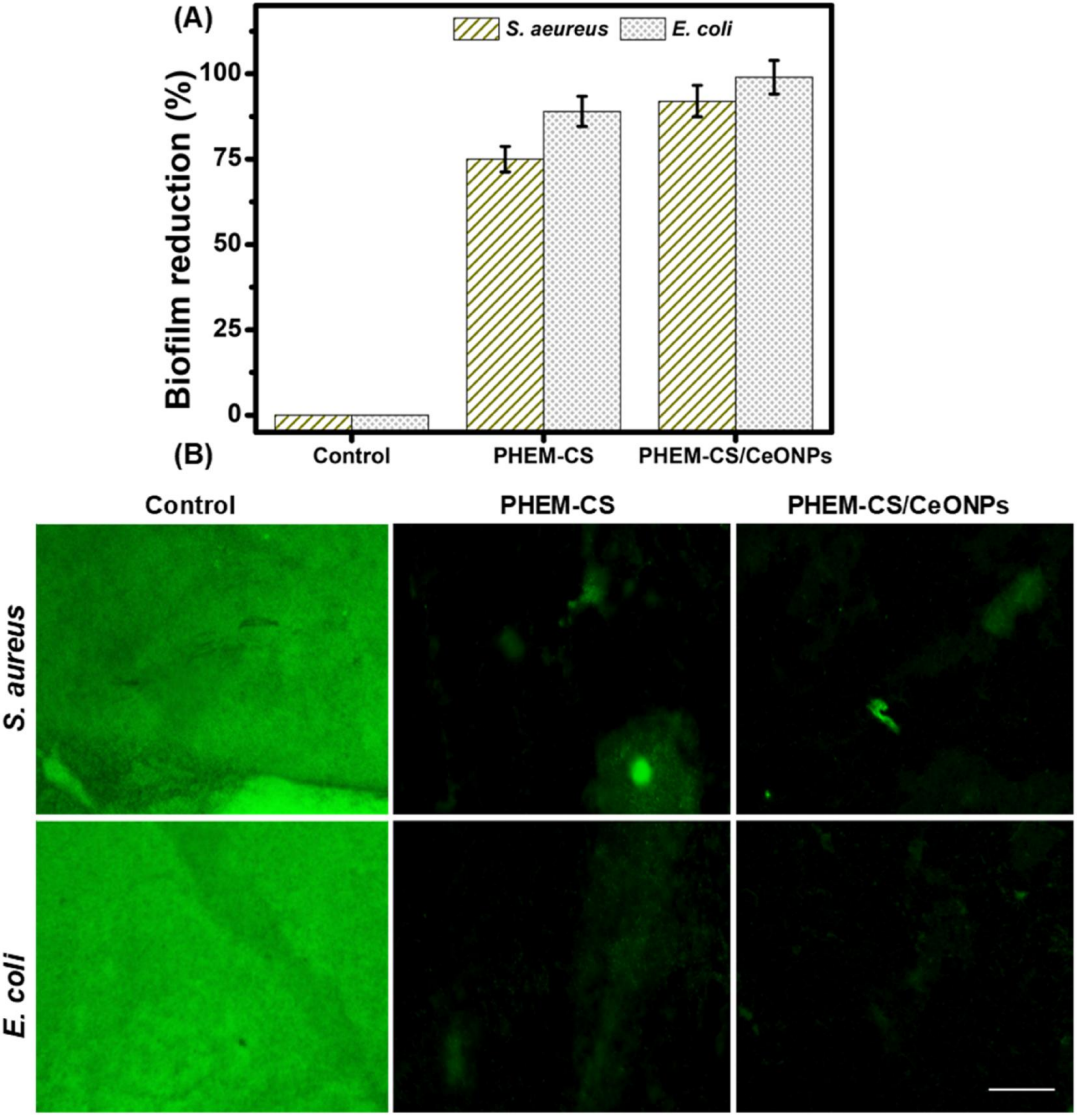
(a) Biofilm reduction and (b) digital images of Control, PHEM‐CS hydrogels, and PHEM‐CS/CeONPs hydrogels nanocomposites. The scale bar is 100 μm. Results are expressed as mean ± SD (*n* = 3), *p* < 0.05. CeONPs, cerium oxide nanoparticles; PHEM‐CS, poly(2‐hydroxyethylmethacrylate)‐chitosan.

One of the primary causes of a sizeable number of human infections has been identified as biofilm development. Recent scientific research has demonstrated that biofilms slow the healing of wounds and increase their susceptibility to infection. It is therefore essential that PHEM‐CS hydrogels and PHEM‐CS/CeONPs hydrogels nanocomposites incorporating antimicrobials demonstrate efficacy on biofilms. The need for improved and more repeatable in vivo biofilm models is crucial. *S. aureus* and *E. coli* were cultivated for several days to form biofilms before the treatment with control, PHEM‐CS hydrogels, and PHEM‐CS/CeONPs hydrogel nanocomposites to investigate the effectiveness of the antimicrobial hydrogels in the elimination of biofilms. CeONPs were loaded at PHEM‐CS into PHEM‐CS/CeONPs hydrogels nanocomposites and placed onto the percentage of biofilm reduction (Figure 6a). The major difference was observed in *E. coli* (Gram‐negative) where cells treated with the more hydrophobic PHEM‐CS/CeONPs hydrogels nanocomposites had significantly lower viability compared to those treated with PHEM‐CS hydrogels. Additionally, CLSM pictures showed that biofilms treated with PHEM‐CS hydrogels and PHEM‐CS/CeONPs hydrogel nanocomposites displayed significant cell clearance and destruction (Figure 6b). For *S. aureus*, only fragments of burst cells remained. The photos also showed strong agreement with quantification studies of biofilm activity, which revealed that PHEM‐CS/CeONPs hydrogel nanocomposites were considerably more efficient at eliminating *E. coli* biofilm than PHEM‐CS hydrogels.

### 3.7 Cytotoxicity of PHEM‐CS/CeONPs hydrogels nanocomposites

**FIGURE 7:**
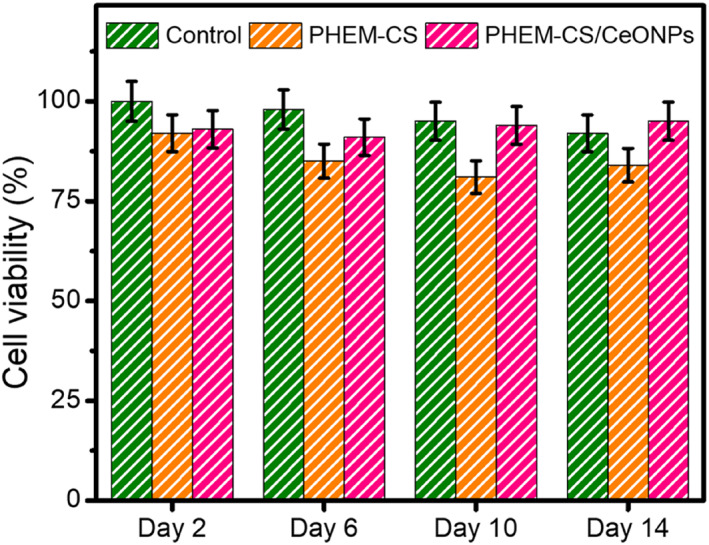
Cytotoxicity of Control, PHEM‐CS hydrogels, and PHEM‐CS/CeONPs hydrogels nanocomposites towards MTT assay using fibroblast (NIH‐3T3) cell lines at days 2, 6, 10, and 14. Results are expressed as mean ± SD (*n* = 3), *p* < 0.05. CeONPs, cerium oxide nanoparticles; PHEM‐CS, poly(2‐hydroxyethylmethacrylate)‐chitosan.

For applications as implants, the PHEM‐CS hydrogels and PHEM‐CS/CeONPs hydrogels nanocomposites must be biocompatible with mammalian cells [45]. Toxic scaffolds, or scaffolds that release toxic byproducts or waste, will kill cells and exacerbate the circumstances in the targeted implant location [46]. Therefore, the toxicities of the PHEM‐CS hydrogels and PHEM‐CS/CeONPs hydrogels nanocomposites were examined by indirect MTT assays using NIH‐3T3 fibroblasts as the model cells. Firstly, the cytotoxicity of PHEM‐CS hydrogels and PHEM‐CS/CeONPs hydrogels nanocomposites were tested by the leaching solution method. As shown in Figure 7, while the fixed concentration of PHEM‐CS/CeONPs hydrogels nanocomposites at 12 mg/mL exhibited more than 90% cell viability compared with PHEM‐CS hydrogels group on days 2, 6, 10, and 14 [47]. A benchmark of non‐toxicity for biomaterial samples is typically cell viability of >70%, according to ISO10993‐5. PHEM‐CS hydrogels groups with a concentration of 12 mg/mL showed significant differences in enhanced cell numbers than the control group (*p* < 0.05). By the 14th day, all of the groups had shown continuous cell proliferation. The in vitro cell test results showed that PHEM‐CS/CeONPs hydrogels nanocomposites had good cell compatibility for NIH‐3T3 fibroblasts [48]. From the results, the fabricated PHEM‐CS/CeONPs hydrogels nanocomposites could potentially recommend a safe drug delivery system.

### 3.8 In vivo evaluation of PHEM‐CS/CeONPs hydrogels nanocomposites

**FIGURE 8:**
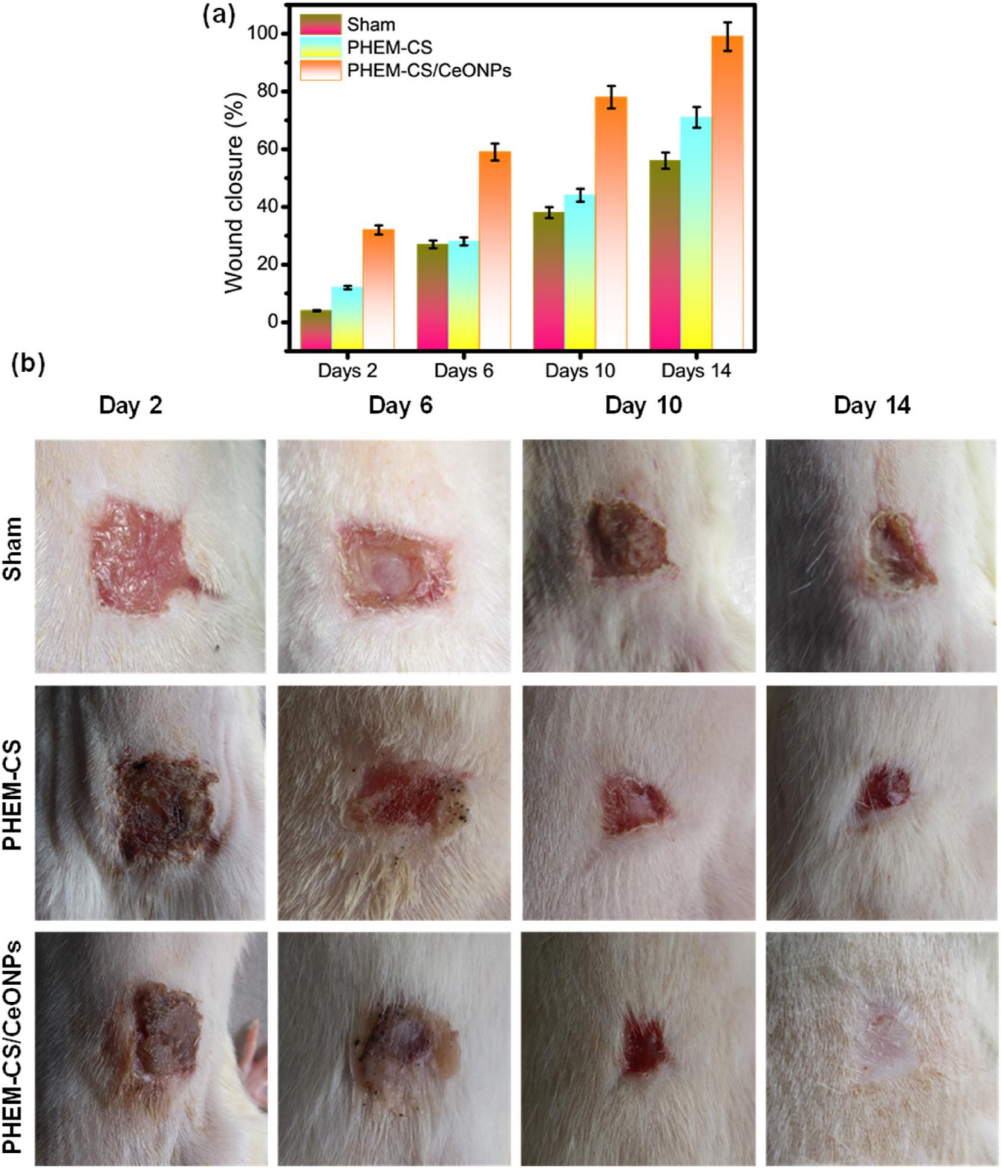
(a) Macroscopic observations (Photo images) of incise wounds at different days (2, 6, 10, and 14) with prepared hydrogel samples and (b) Representative photographs of full‐thickness excision wounds at different time intervals at days 2, 6, 10, and 14 following treatment with Sham group, PHEM‐CS hydrogels and PHEM‐CS/CeONPs hydrogels nanocomposites treated groups. Results are expressed as mean ± SD (*n* = 3), *p* < 0.05. CeONPs, cerium oxide nanoparticles; PHEM‐CS, poly(2‐hydroxyethylmethacrylate)‐chitosan.

The in vivo wound healing studies were carried out in Sprague‐Dawley rats, and the corresponding results were presented in Figure 8. According to current studies, the addition of CeONPs to PHEM‐CS hydrogels leads to improved wound healing, providing unique antibacterial properties due to their excellent mechanical and optical properties. A full‐thickness wound (1 × 1 cm^2^) was formed on the back of each rat in our wound healing rat model, and the wound healing process was measured using the wound closure ratio on the 2, 6, 10, and 14 postoperative days. The wound contraction for the Sham group was found to be 4 ± 0.9%, 27 ± 2.1%, 38 ± 1.8%, and 56 ± 1.2% on 2, 6, 10, and 14 days. The wound contraction for the PHEM‐CS hydrogels group was found to be 12 ± 0.6%, 28 ± 1.4%, 44 ± 2.2%, and 71 ± 3.55% on 2, 6, 10, and 14 days. The PHEM‐CS/CeONPs hydrogels nanocomposites group showed contraction on 32 ± 1.6%, 59 ± 2.95%, 78 ± 3.9%, and 98.5 ± 4.95% on 2, 6, 10, and 14 days (Figure 8a). The wounds treated with PHEM‐CS/CeONPs hydrogels nanocomposites dressings showed a better healing effect than that of the wounds without any treatment (the control; sham group) or the wounds treated with PHEM‐CS hydrogels, indicating that the obtained novel hydrogel wound dressings were beneficial for accelerating the wound healing [49]. Furthermore, as demonstrated in Figure 8a, CeONPs incorporating PHEM‐CS hydrogels had a substantially faster complete wound healing (re‐epithelialisation) than the other treatment groups. Images captured with a camera, as shown in Figure 8b, corroborate these findings. Thus the Sprague‐Dawley rats treated with the PHEM‐CS/CeONPs hydrogels nanocomposites showed less postoperative infection than the PHEM‐CS hydrogels groups and recovered earlier than the others.

### 3.9 Histological analysis of PHEM‐CS/CeONPs hydrogels nanocomposites

**FIGURE 9:**
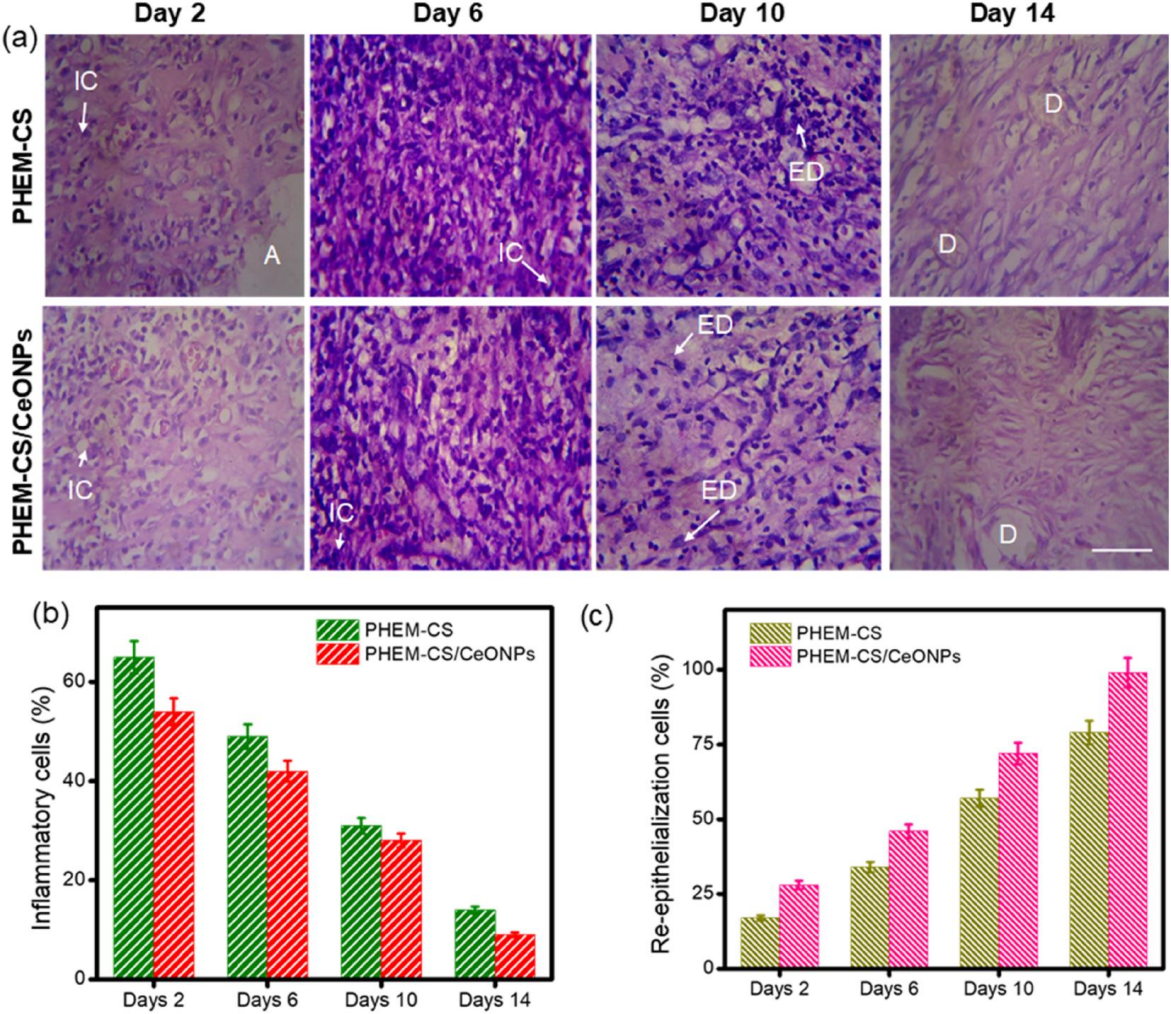
Haematoxylin and eosin stained sections of the granulation tissue of PHEM‐CS hydrogels and PHEM‐CS/CeONPs hydrogels nanocomposites treated groups at different time intervals at days 2, 6, 10, and 14. Scale bar ∼2 μm. (IC, inflammatory cells; A, ulceration; ED, epidermis; D, dermis); (b) Histogram of inflammatory cells and (c) re‐epithelialisation in percentage values. Results are expressed as mean ± SD (*n* = 3), *p* < 0.05. CeONPs, cerium oxide nanoparticles; PHEM‐CS, poly(2‐hydroxyethylmethacrylate)‐chitosan.

To assess the wound healing impact at various phases, haematoxylin and eosin‐stained sections (H&E staining) were used. In comparison to PHEM‐CS hydrogels, CeONPs included PHEM‐CS hydrogels showed scar formation with considerable wound contraction, improved re‐epithelialisation, and considerable keratinocyte migration, whereas PHEM‐CS hydrogels showed scar and scab development with reduced wound contraction [50] (Figure 9). The wounds were studied by examining the histology at days 2, 6, 10, and 14 postoperative days. PHEM‐CS hydrogels treated group showed good proliferation of cells in the wounded area and less scar formation [51, 52]. In the combined therapy group, moderate granulation tissues with a few inflammatories infiltrates and considerable epidermal gain was seen. All groups had complete epidermal coverage over the wound surface on the 14th day after injury. PHEM‐CS/CeONPs hydrogels nanocomposites also showed proliferation and rudimentary hair follicles. Furthermore, PHEM‐CS/CeONPs hydrogels nanocomposites demonstrated superior rudimentary hair follicle production. CeONPs were found to be important because they included PHEM‐CS hydrogels in the dressings, which were known to reduce inflammation at the wound site [26].

When compared to the untreated group, the histopathological appearance of the burning skin epithelium changed significantly, and the burning epithelium treated with PHEM‐CS/CeONPs hydrogels nanocomposites indicated statistically substantial restoration of the skin against burn‐induced skin damage, especially in the burn‐induced damage after cerium treatment [53, 54]. It was observed that PHEM‐CS/CeONPs hydrogel nanocomposites treated wounds had a higher initial rate of re‐epithelialisation (Figure 9b) than the PHEM‐CS hydrogels group. In this study, CeONPs activated bioactive hydrogel treatment was active in accelerating wound healing up to 14 days after treatment. Aside from that, the PHEM‐CS/CeONPs hydrogels nanocomposites were found to reduce inflammatory cell activity to 72 ± 1.2% and 98 ± 0.9% (Figure 9c), which was better than PHEM‐CS hydrogels. This indicates that PHEM‐CS/CeONPs hydrogels nanocomposites are effective at controlling inflammation by their combined anti‐inflammatory effect. These results proved that PHEM‐CS/CeONPs hydrogels nanocomposites had the potential to be applied as wound dressings.

## 4 CONCLUSIONS

The PHEM‐CS hydrogels and PHEM‐CS/CeONPs hydrogels nanocomposites were prepared via cross‐linking reaction. These hydrogels showed short gelation time, high swelling ratio, excellent in vitro degradation, and stable mechanical properties, which can effectively promote wound healing. The porous network structure formed after cross‐linking has been confirmed from the SEM images of the PHEM‐CS/CeONPs hydrogels nanocomposites dressings. Furthermore, the PHEM‐CS/CeONPs hydrogels nanocomposites showed excellent antibacterial properties against both *S. aureus* and *E. coli* bacteria as demonstrated by the inhibition zone test as well as biofilm activity. Furthermore, the PHEM‐CS/CeONPs hydrogels nanocomposites accessible good biocompatibility by the MTT test. Histopathological evidence indicates that PHEM‐CS/CeONPs hydrogels nanocomposites were more active than PHEM‐CS hydrogels with burn healing on rats for 14 days. These PHEM‐CS/CeONPs hydrogels nanocomposites are promising materials for wound healing and tissue engineering.

## AUTHOR CONTRIBUTIONS

**Qiaoling Xie**: Formal analysis; Resources; Software. **Jianshu He**: Project administration; Supervision. **Liyan Jiang**: Investigation; Writing – original draft; Writing – review & editing.

## CONFLICT OF INTEREST STATEMENT

The authors declare that they have no known competing financial interests or personal relationships that could have appeared to influence the work reported in this paper. There is no potential conflict of interest.

## PERMISSION TO REPRODUCE MATERIALS FROM OTHER SOURCES

None.

## Data Availability

The data that support the findings of this study are available from the corresponding author upon reasonable request.
